# Pilocarpine suppresses hyperalgesia induced by intermittent cold stress (ICS) as an experimental fibromyalgia model in mice

**DOI:** 10.1186/ar3652

**Published:** 2012-02-09

**Authors:** Jun Nagai, Michiko Nishiyori, Hiroshi Ueda

**Affiliations:** 1 Division of Molecular Pharmacology and Neurosciences, Nagasaki University Graduate School of Biomedical Sciences, Nagasaki 852-8521, Japan

## 

Fibromyalgia (FM) is a highly populated chronic pain disease, which has unique characteristics including generalized or widespread allodynia and female prevalence of gender difference. Many FM patients are common with Sjögren's syndrome. Pilocarpine, a non-selective muscarinic receptor agonist, is used clinically as a drug that promptes the secretion of salvia for dry eyes and mouth. Otherwise, pilocarpine has been shown to possess antinociceptive effect, which maybe caused by vagal afferents activation. The experimental FM mice exposed to intermittent cold stress (ICS) showed sustained abnormal pain, such as mechanical allodynia and hyperalgesia to nociceptive thermal stimuli for up to 19 days, but those given constant cold stress (CCS) did not. The abnormal pain was bilateral (generalized), female-predominant and specific for A-delta and A-beta, but not C-fiber-stimuli. In ICS mice, intraperitoneal or oral administration of pilocarpine showed potent anti-hyperalgesic effects in doses without excess salivation at post-stress day5 (P5). The anti-hyperagesic effects last for more than 1 h, but disappear at 24 h. Daily administration of pilocarpine showed equivalent anti-hyperalgesic effects without tolerance. These findings suggest that pilocarpine possesses a beneficial effect for the pain treatment of FM patients with dry eyes and mouth symptoms.
